# Editorial: Sleep, vigilance & disruptive behaviors

**DOI:** 10.3389/fpsyt.2023.1230825

**Published:** 2023-07-21

**Authors:** Osman S. Ipsiroglu, Gerhard Klösch, Rosalia Cesarea Silvestri, Susan Mary McCabe, Georg Dorffner, Thomas Christian Wetter, Luci Wiggs

**Affiliations:** ^1^Departments of Pediatrics and Psychiatry, University of British Columbia, Vancouver, BC, Canada; ^2^Department of Neurology, Medical University of Vienna, Vienna, Austria; ^3^Centro Interdipartimentale per la Medicina del Sonno UOSD di Neurofisiopatologia e Disordini del Movimento, Dipartimento di Medicina Clinica e Sperimentale, Messina, Italy; ^4^Edith Cowan University, Joondalup, WA, Australia; ^5^Centre for Artificial Intelligence and Decision Support, Medical University of Vienna, Vienna, Austria; ^6^Regensburg Center of Neuroscience, University of Regensburg, Regensburg, Bavaria, Germany; ^7^Centre for Psychological Research, Oxford Brookes University, Oxford, United Kingdom

**Keywords:** developmental pediatrics, mental health, child and adolescent psychiatry, convention on the rights of the child, article 24 - the rights of the child, sleep, vigilance, disruptive behaviors

The Frontiers in Psychiatry Research Theme of *Sleep, vigilance, and disruptive behaviors* has two aims: first, to promote the understanding of the connections between vigilance and disruptive daytime behavior in the context of sleep deprivation and, second, to explore how naturalistic observations and pattern recognition can play a role in furthering our understanding of these connections. The theme is devoted to the British neurologist Henry Head and German psychiatrist Heinrich Hoffmann. In 1923, Head defined vigilance as the ability of the body “to respond to an effective stimulus with a more or less appropriate reaction” (1). In 1845, Hoffmann, at that time still a general practitioner, depicted the connection of “vigilance” with disruptive behaviors of children and youth in a cartoon book entitled, “Struwwelpeter—Merry Tales and Funny Pictures” [(2, 3) and see Figure 1 for book cover]. In this book, each story describes a clinical condition and the multiple links to affected vigilance, which in consequence cause disruptive daytime behaviors. The narratives of “Fidgety Philip” (for attention deficit hyperactivity disorder, ADHD and/or restless legs syndrome, RLS), “John Head-in-Air” (attention deficit disorder paired with sensory processing dysfunctions), “Struwwelpeter” and “Conrad” (sensory processing dysfunctions paired with obsessive compulsive behaviors), “Augustus” (eating disorders), “Flying Robert” and “Harriet” (oppositional defiant disorder), “The Inky Boys” (mobbing and bullying in context with racism), and “Cruel Frederick” (oppositional defiant disorder) were originated from observations utilizing pattern recognition and are described in an easily understandable visual format of cartoons. Both impaired vigilance and disruptive behaviors mirror sleep disturbances. They can also be observed and are independent of cultural norms, enabling an exploratory screening approach to an individuals' wellbeing. Importantly, these are commonly in conjunction with underlying and missed sleep problems, which have been culturally segregated and medicalised areas—usually investigated by daytime-focused sub-specialists, without acknowledging sleep disturbances. Indeed, naturalistic observations and their meaning are the foundation of clinical history taking, a practice commonly used in communication between clinicians and patients (including parents in the case of pediatric patients). A discourse to examine the validity of outsourcing such observable contextual concepts into lab settings without capturing and agreeing first on the observed patterns, their epistemic justification, and phenomenology is overdue. The Research Theme *Sleep, vigilance, and disruptive behaviors* is a Frontiers in Psychiatry eBook, consisting of 14 peer-reviewed articles and discussing a variety of topics such as sleep and how it affects daily functioning, the broadest definition of vigilance, and behaviors seen as disruptive in children and as challenging or worrisome in adults. All articles, whether they are reviews, case reports, or cohort studies, invite the readers to review the interconnections among the three topics *via* naturalistic observations and exploration from multiple perspectives.

**Figure 1:**
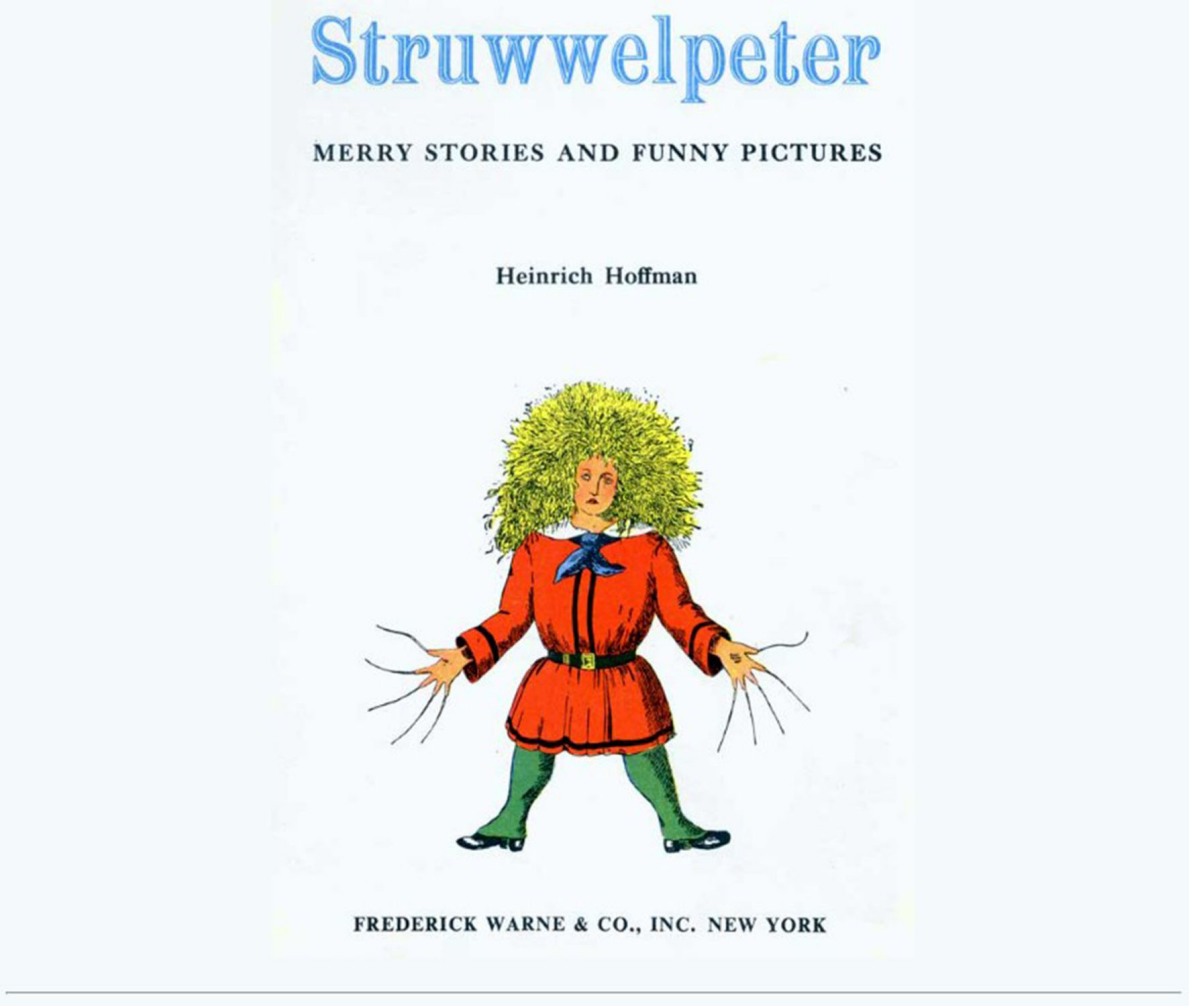
“Just look at him! there he stands, With his nasty hair and hands. See! his nails are never cut; They are grimed as black as soot; And the sloven, I declare, Never once has combed his hair; Anything to me is sweeter Than to see Shock-headed Peter.” (3).

Of the five review articles, the first one revisits the *concept of vigilance* as an indicator of sleep disturbances. Since the introduction of the vigilance concept by Head, many different aspects were analyzed; however, the underlying context for gathering insight into the interplay between sleep and daytime behaviors, reflecting both physical and cognitive performance, as well as sleep quality and quantity, was hardly ever investigated (Klösch et al.). Similarly, the second review article explores a *universal screening strategy for sleep health in the community*, utilizing social-ecological considerations, as the cultural context of the current (medicalised) approach does not acknowledge the myriad of presentations and possible root causes of sleep disturbances (Blunden et al.). The third review examines the functional links between *thermoregulation for maintaining thermoneutrality and sleep* in children with chronic health conditions as circadian patterns of sleep-wake are dependent on patterns of body temperature changes (McCabe et al.). The fourth review and meta-analysis investigates the efficacy of *eye masks and earplugs in intensive care units* as an intervention for promoting sleep health (Karimi et al.). The fifth review investigates the root causes of most hypermotor restlessness, such as central iron deficiency and its exacerbation by vitamin D deficiency (Silvestri and Ipsiroglu). While the first two reviews focus on screening and how to integrate a sleep screening in a tier service model (Blunden et al.; Klösch et al.), the latter three reviews demonstrate how, with minimal consideration, sleep health can be promoted in the various facets of modern medicine and mirrors the need for a holistic approach to sleep and sleep health and how harmonization of first-line treatment options could improve sleep health (Karimi et al.; McCabe et al.; Silvestri and Ipsiroglu).

The second block consists of six articles investigating the associations between sleep and behavioral patterns, e.g., ADHD, utilizing big and small data. Vigilance regulation disturbances in the awake state play a key role in the development of mental health disorders. Hyperactivity in ADHD is an attempt to increase the low vigilance level *via* external stimulation to avoid drowsiness—this common hypothesis led to the *analysis of resting-state EEGs in children diagnosed with ADHD or depression* (Berger et al.). The study, using longitudinal “big” data from the National Korean Registry, suggests that addressing *underlying sleep disturbances rather than sleep duration* is the most important factor in predicting and preventing adjustment problems in young children. Furthermore, more attention should be paid to maternal depressive symptoms in preschooler years as much as during the postpartum period for better child adjustment outcomes (Cha). The next two studies utilized small data and a qualitative approach. While one study explores *disruptive behaviors of adolescents with Down syndrome* in a summer school setting, including the link to probable familial RLS, relieved by hours of physical activity, this qualitative study reveals that disruptive behaviors of children with intellectual disabilities have different connotations depending on guiding contextual frameworks (Chan et al.). Finally, yet importantly, in a qualitative study, *parental challenges in sourcing effective sleep solutions for their child with cerebral palsy* are explored. Sleep may be a low priority for parents or clinicians as other health problems take precedence (Petersen et al.). *The RLS prevalence in hospitalized psychiatric patients, a multicenter adult study from Germany and Switzerland*, rounds this picture. Clinically significant RLS had almost five times higher prevalence in psychiatric patients, and more than three-quarters were diagnosed with RLS for the first time, which necessitates for an RLS screening (Weber et al.). The brief research report on *patient characteristics and medication prescriptions in children with mental health and neurodevelopmental disorders referred to a Canadian sleep clinic* demonstrates that special attention to probable RLS-induced insomnia should be given as early as the triaging process at the community level (Ipsiroglu et al.). Note that RLS, even familial RLS, is an underestimated clinical sleep/wake-behavioral diagnosis, where there is no need for a sleep laboratory-based diagnostics—instead, relying solely on naturalistic observations and exploration, including structured history taking and blood work, steps which should become essential in assessments of insomnia [Silvestri and Ipsiroglu, (4)].

The third block analyses exposure to sleep/wake behaviors and timing, in relation to digital media. In a large study from the United Kingdom, the relationship between *smartphone addiction and sleep quality in young adults* was investigated demonstrating that 39% of young adults reported smartphone addiction. Smartphone addiction was associated with poor sleep, independent of the duration of usage, indicating that the length of time should not be used as a proxy for harmful usage (Sohn et al.). Data from South Germany, a region with high social-economic status, show that the actual *exposure to digital media may start already in 12-month-old infants*; a proportion of 10% of 1-year-old children was already regularly exposed to digital media (Durham et al.). Given the warnings of the American Academy of Pediatrics and national guidelines, which recommend no digital media use at all under the age of 18 months, the question could be around how this rate might fluctuate in varied regions with different social-economic statuses. Explorations for *understanding sleep-wake behaviors in late chronotype adolescents* show that with increasing lateness, the likelihood of experiencing poor sleep quality and mood disorders increases (Lang et al.). However, bedtime was not predicted by dim light melatonin onset (DLMO) indicating that the factors contributing to a late chronotype are multiple and complex. Understanding these contributing factors, and their relative importance across individuals, needs exploration. Again, this article proves our leitmotif that naturalistic observations and exploration will open up new perspectives to typical “disruptive” adolescent behaviors.

Reading these articles, as an editorial team, we have been thinking about critical issues in our field. Sleep is an important public health issue. However, the current emphasis is on clinical sleep medicine as a Western-centric urban sub-specialty, where we have not implemented a universal screening concept for sleep health, and the knowledge regarding pattern recognition (see Head's *vigilance concept* and Hoffman's *disruptive behaviors)* is often overlooked, or even unknown. Many partners in the community, such as public health nurses, occupational therapists, psychologists, general practitioners, internists, psychiatrists, and even pediatricians and child and adolescent psychiatrists, lack basic sleep health training and knowledge. Thus, we all unanimously agree to advocate for establishing sleep as a priority on the national public health agenda. We suggest “*HumanRight2Sleep”'* or “*ChildRight2Sleep”* as the communication motto for overcoming a checklist-based daytime focus, a *rights-based approach to sleep disturbances* may support us to review things from a patient rather than a professional sub-specialist perspective and move the agenda further.

## Author contributions

All authors listed have made a substantial, direct, and intellectual contribution to the work and approved it for publication.
